# The vagal rhizopathies

**DOI:** 10.3389/fneur.2024.1513160

**Published:** 2025-01-22

**Authors:** Christopher R. Honey

**Affiliations:** Professor and Head, Division of Neurosurgery, University of British Columbia, Vancouver, BC, Canada

**Keywords:** microvascular decompression, vagus nerve (CN X), vagal rhizopathy, hemi-laryngopharyngeal spasm, VANCOUVER syndrome, vagal neuralgia, neurogenic cough

## Abstract

Neurovascular compression of the tenth cranial nerve, the vagus nerve, can cause recognizable and neurosurgically treatable clinical conditions. This chapter will outline the clinical characteristics unique to vagus nerve compression and highlight both the definitive diagnostic protocol and neurosurgical treatment of these conditions. The vagus nerve has motor, sensory and autonomic components. Neurovascular compression of the motor component can cause hemi-laryngopharyngeal spasm (HELPS syndrome). Compression of the sensory component will cause a neurogenic cough called VANCOUVER syndrome – an acronym for Vagus Associated Neurogenic Cough Occurring due to Unilateral Vascular Compression of its Root. Both are caused by direct compression of the root of the tenth cranial nerve at the brainstem by a blood vessel and can be cured by microvascular decompression (MVD). Since the symptoms of choking and cough are common and blood vessels are often abutting the vagus nerve at the brainstem, it is vitally important to understand the definitive diagnostic protocol to avoid operating on false positives. Since the vagus nerve is far more susceptible to dysfunction during surgery than either the trigeminal or facial nerves, it is also important to understand the surgical nuances of this procedure.

## Introduction

Vascular compression of the 5th cranial nerve (trigeminal nerve) and 7th cranial nerve (facial nerve) can cause the classic features of trigeminal neuralgia and hemifacial spasm, respectively. This chapter describes the clinical features of a vascular compression of the 10th cranial nerve (vagus nerve). The vagus nerve has motor, sensory and autonomic components. The clinical features associated with a vascular compression of the motor and the sensory components can each be distinguished and have been previously published. The two resultant conditions are known as hemi-laryngopharyngeal spasm (HELPS syndrome) (1) and vagus associated neurogenic cough occurring due to unilateral vascular encroachment of its root (VANCOUVER syndrome) (2).

### Hemi-laryngopharyngeal spasm (HELPS syndrome)

The motor fibers of the vagus nerve innervate the muscles of the pharynx and larynx. A vascular compression of this component of the vagus nerve can cause intermittent contractions of these muscles. This is similar to the hemifacial spasm that results from compression of the motor fibers within the facial nerve. The condition can be diagnosed by a combination of history, physical examination and special tests (1, 3, 4).

#### History

When the muscles of the pharynx are involved, the patient can localize the contractions to one side of their throat. When the muscles of the larynx are involved, the patient describes a non-lateralized general constriction of their airway. If both pharyngeal and laryngeal muscles are involved, the patient can lateralize their symptoms. The contractions are intermittent and patients are entirely normal between episodes. The contractions can last seconds to minutes and be very distressing for patients who can report difficulty breathing. There is no associated pain unless the condition has a concurrent glossopharyngeal neuralgia due to compression of the adjacent 9th cranial nerve (glossopharyngeal nerve) (5). The condition will typically progress over the years with the attacks becoming more frequent, lasting longer and severe. A unique aspect (also found in hemifacial spasm) is that the attacks of muscle contractions can occur while asleep.

The intermittent muscle spasms are associated with a cough. The cough is due to compression of the sensory fibers of the vagus nerve and will be described in detail below under VANCOUVER syndrome. We have never seen a patient with pure hemi-laryngopharyngeal spasm without a cough but one patient reported the motor contractions started before any coughing. We have seen patients with pure cough (see VANCOUVER syndrome below) without the muscles spasms of hemi-laryngopharyngeal spasm. Severe episodes of this coughing can produce unconsciousness. The likely etiology for unconsciousness is cough syncope rather than airway obstruction.

Patients can report other episodic symptoms due to contractions of muscles innervated by the vagus nerve. An intermittent “fat” tongue sensation may be due to pallatoglossus contractions; episodic vocal changes triggered by prolonged or loud talking may be due to intrinsic laryngeal muscle contractions and a globus sensation may be due to pharyngeal constrictors contraction.

The symptoms are refractory to the usual medications and treatments prescribed for episodic laryngospasm or chronic cough. Patients do not respond to proton pump blockers, speech therapy, psychotherapy, bronchodilators or antibiotics. The muscle contractions can be temporarily stopped by Botox injection into the correct muscles but will typically not respond to anti-neuralgia medications (like hemifacial spasm). The cough, however, may respond to anti-neuralgia medications (like trigeminal neuralgia).

#### Physical examination

In between episodes, the patient’s examination is entirely normal. This often leads to the misdiagnosis of a psychogenic disorder. The patient’s unpredictable, severe symptoms and typical difficulty with obtaining a diagnosis can lead to anxiety, depression and hostility toward medical workers thereby strengthening the psychogenic misdiagnosis.

During an attack, the patient is in extremus and fighting to breath. This may explain why there has not yet been a laryngoscopy reported during an attack. A portion of patients (approximately one-third) have a pathognomonic movement disorder of their vocal folds (4). This has been reported as a unilateral vocal fold twitch following vocalization. It is unique to hemi-laryngopharyngeal spasm and resolves following surgical cure. There are no other physical features between episodes remarkable for this condition except for the absence of any signs pointing to an alternate diagnosis.

#### Special tests

**Figure 1:**
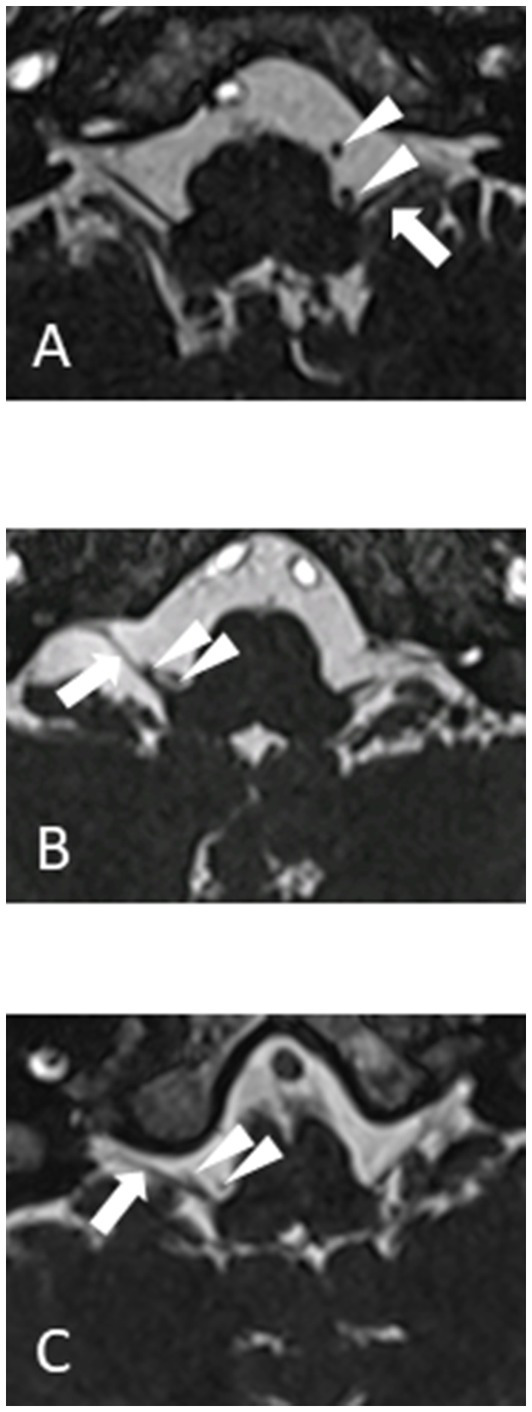
1.5 T MRI CISS sequences of three different patients **(A, B** and **C)** showing vagus nerve (arrow) and loop of PICA (arrowheads).

MRI must demonstrate a vascular compression of one of the vagus nerves. Imaging sequences are the same as for the other neurovascular compression syndromes (CISS or FIESTA). Care must be taken not to over diagnose the condition because asymptomatic patients can have a vessel on their vagus nerve up to 40% of the time (6). An MRI documented compression of the vagus nerve is therefore required but not sufficient for the diagnosis of hemi-laryngopharyngeal spasm. The compressing vessel is typically the posterior inferior cerebellar artery (PICA) with a 180-degree loop deflecting the vagus nerve. Three examples are shown in Figure 1. The vagus nerve rootlets are small enough to make their individual resolution difficult even with high field 3 T MRI. The looping PICA may show as two flow voids on a 2-dimentional axial plane of the MRI.

Laryngoscopy may show a pathognomonic ipsilateral twitch of the vocal fold following vocalization. An example has been published in the otolaryngology literature (4). This movement disorder appears to be unique to this condition and has not been previously reported. Unfortunately, not all patients demonstrate this finding. When it is present, however, it definitively points to the affected side.

Botulinum toxin (Botox) injected into the affected muscles will stop (or dramatically improve) the intermittent contractions but not the cough. Botox injected into the contralateral side will have no beneficial effect.

#### Diagnostic protocol

**Figure 2:**
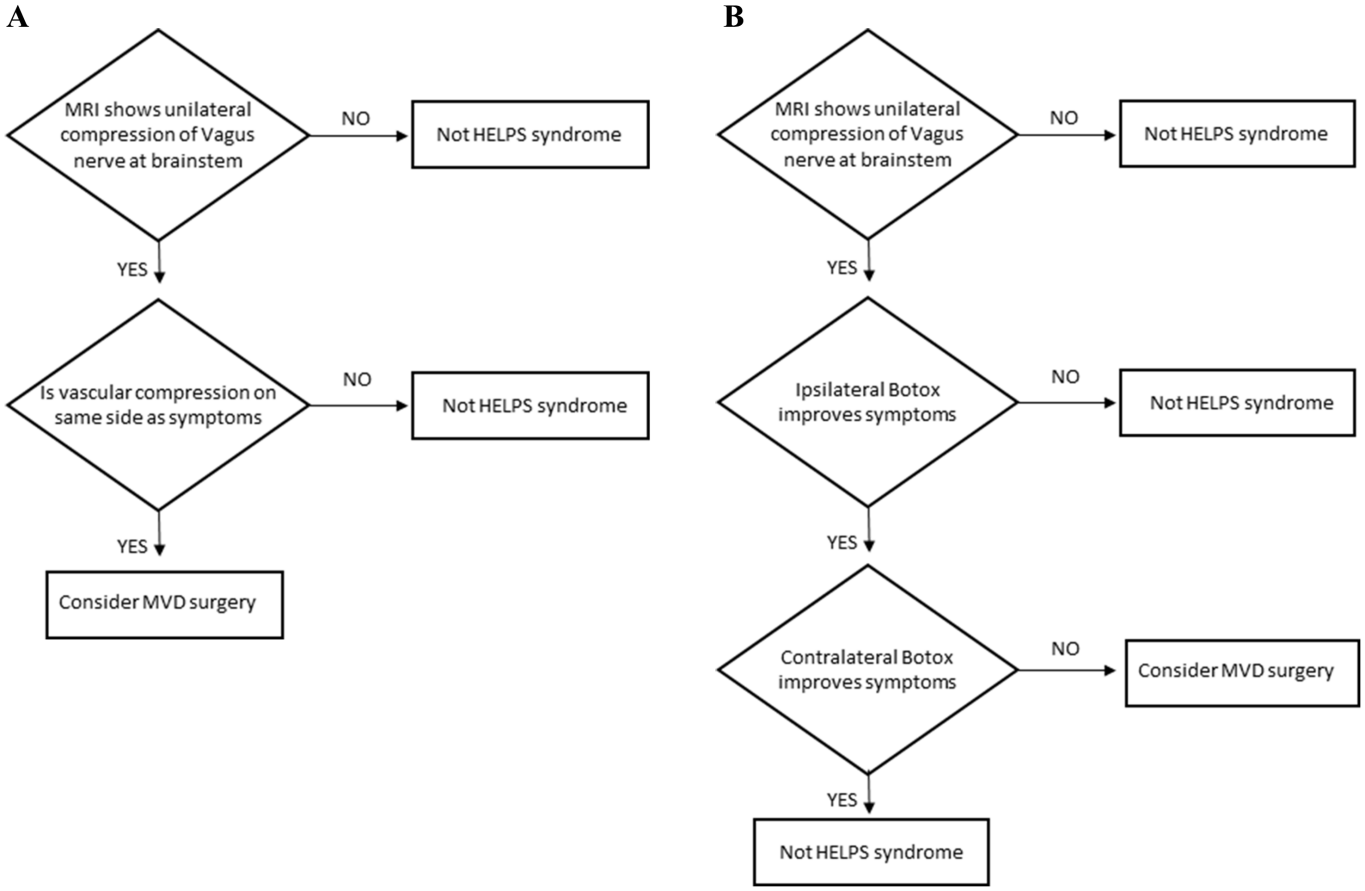
**(A)** Diagnostic protocol for hemi-laryngopharyngeal spasm in patients who can lateralize their symptoms. **(B)** Diagnostic protocol for hemi-laryngopharyngeal spasm in patients who cannot lateralize their symptoms.

Patients with chronic, intermittent, medically refractory throat contractions and cough should be tested for a diagnosis of hemi-laryngopharyngeal spasm. The diagnostic protocol for patients who can lateralize their symptoms is shown in Figure 2A. If the MRI confirms a vascular compression on the same side as their symptoms, consideration can be given to offering microvascular decompression of the vagus nerve. The diagnostic protocol for patients who cannot lateralize their symptoms is shown in Figure 2B. Since those patients can not tell which side is affected (usually due to laryngeal muscles causing a circumferential sensation of choking) the beneficial effects of Botox must be used to locate the affected side. When Botox is injected into the symptomatic side, the episodic muscle contractions will be greatly diminished (typically 80% improved). The procedure is later repeated (once the effects of Botox have worn off after 3 months or more) on the contralateral side to confirm this is a unilateral benefit. The patient should have no benefit from the contralateral Botox. If the patient does improve with the contralateral Botox, then the condition is generalized, not unilateral, and can not be hemi-laryngopharyngeal spasm. There might be a theoretic patient with bilateral symptoms but that must be exceedingly rare (like bilateral hemifacial spasm).

#### Medical therapy

The motor contractions will be diminished with repeated Botox therapy into the affected muscles. This is similar to hemifacial spasm. The side effects of Botox therapy are related to the targeted muscles and include dysphagia and dysphonia. The cough may respond to anti-neuralgia medications such as Carbamazepine or Neurontin. The side effects of these medications are well described for trigeminal neuralgia and can include rash, sedation or cognitive slowing.

#### Surgical therapy

**Figure 3:**
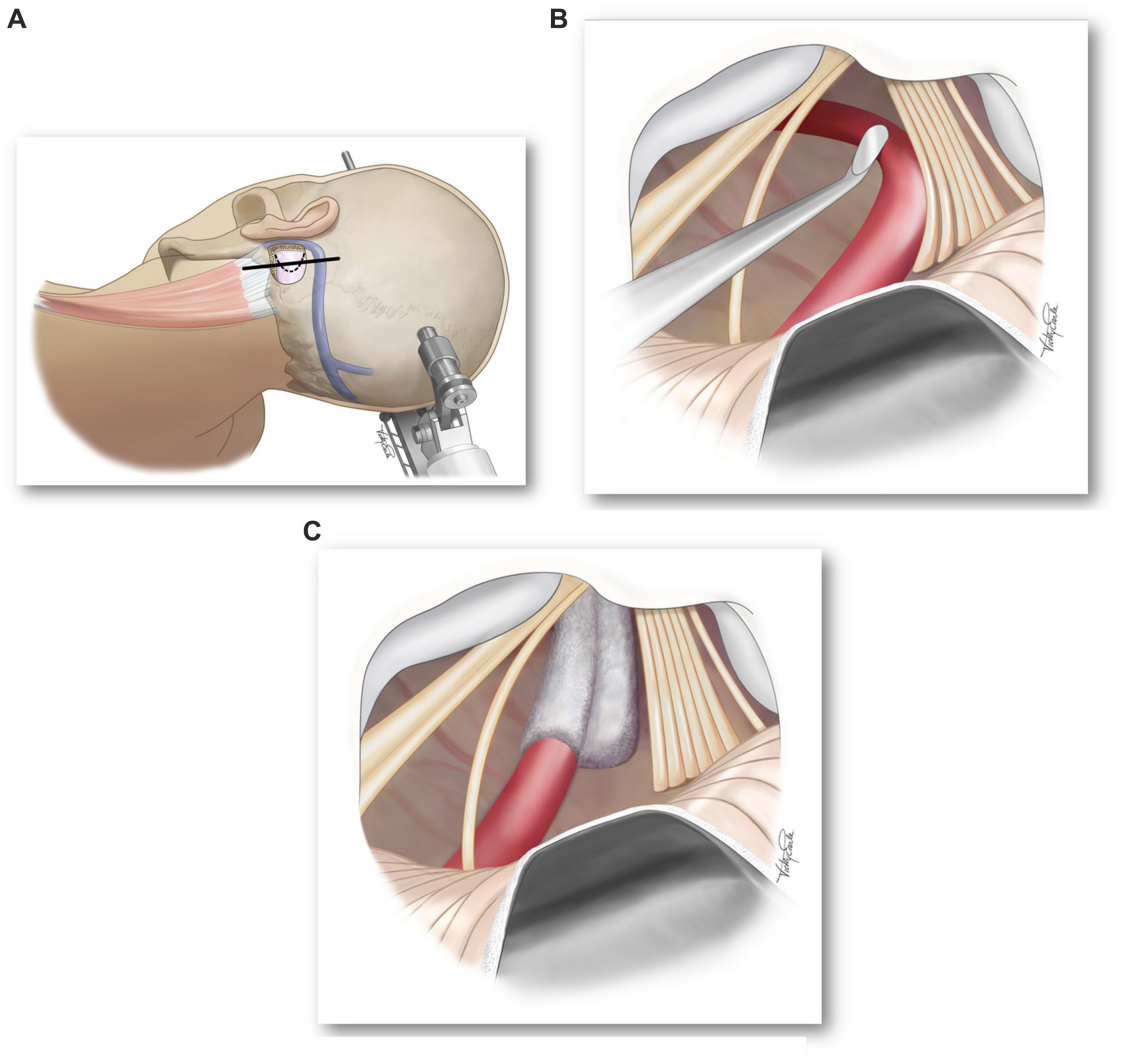
**(A)** Surgical position and location of skin (solid line) and dural (dotted line) incisions. **(B)** Microscope image showing compression of vagus nerve rootlets by a loop of the PICA. **(C)** Microscope image showing decompression of vagus nerve rootlets.

The definitive therapy for hemi-laryngopharyngeal spasm is microvascular decompression of the vagus nerve. The surgical approach is similar to that for exposing the 7th cranial nerve (hemifacial spasm) or the 9th cranial nerve (glossopharyngeal neuralgia). The most important aspect of the surgical approach is that the surgeon is familiar and comfortable with it. Our team has used the lateral position with the affected side up to facilitate the cerebellum falling away from the inside of the occipital bone (Figure 3A). Additional brain relaxation is facilitated with hyperventilation (PaCO_2_ = 30 mmHg). The patient will have neuromonitoring of their eighth cranial nerve. The procedure begins using surgical loops and a retrosigmoid incision. The craniectomy is extended inferiorly to the horizontal portion of the occipital bone and laterally to the medial edge of the sigmoid sinus (the transverse sinus does not need to be exposed). The craniectomy is then widened superiorly and medially to permit easy access of instruments intradurally. The dura is opened in a curvilinear fashion, convex medially, with the base laterally toward the sigmoid sinus and turned to expose the cerebellum.

The operating microscope is then brought into play. After cerebrospinal fluid (CSF) is drained, the cerebellum can be mobilized medially and superiorly exposing the lateral wall of the posterior fossa. The exposure is slowly widened until the arachnoid over the lower cranial nerves is exposed. The arachnoid is then sharply dissected and additional CSF will be released. The spinal root of the 11th cranial nerve (accessory nerve) can be seen curving superiorly to join the multiple rootlets of the vagus nerve entering the jugular foramen. There are typically five or six separate rootlets of the vagus and then just rostral will be a single root (typically larger and whiter) of the 9th cranial nerve (glossopharyngeal nerve).

At this point, the vascular compression of the vagus nerve will be apparent (Figure 3B) and a decision should be made as to how best to move the vessel away and maintain its decompression (Figure 3C). The vagus nerve does not tolerate as much manipulation as the trigeminal nerve and is even more sensitive than the facial nerve. Excessive manipulation will cause post-operative dysphagia and dysphonia. Care should also be taken not to manipulate or compress the cranial root of the 11th cranial nerve as it controls laryngeal muscles and can result in an ipsilateral vocal fold paralysis. We prefer not to have any residual compression of the vagus nerve – not by the vessel or the Teflon pad.

The dura is closed watertight and methyl methacrylate used to refashion the craniectomy. The scalp is closed with running absorbable suture (2–0 Vicryl) in the fascia and a watertight running locked nonabsorbable suture (3–0 Proline) in the skin. Extubation is done with care to avoid excessive Valsalva coughing.

Post-operatively, the patient’s ability to swallow must be confirmed before they are allowed to be fed. Temporary dysphagia can occur and patients may need to be tube fed until it resolves. The function of the patient’s vocal folds can be assessed at the bedside by listening to their voice. A soft, raspy voice may indication vocal fold dysfunction.

The benefits are typically seen immediately but, like hemifacial spasm, may require some time to resolve. Our small but growing surgical experience with this condition has been published (4). Six patients have had more than five-years follow-up and five remain symptom free. The sixth patient had resolution of their cough but incomplete improvement of their choking. One patient developed dysphagia four years post-operative and had repeat MVD to decompress the Teflon off the lowest rootlet of the vagus nerve. Their dysphagia resolved (see Future Issues below). Two patients had post-operative ipsilateral vocal fold paralysis. These two had complete glottic closure and denied dysphonia, dysphagia or aspiration. One patient had temporary dysphagia with complete resolution by two months and was able to have their pre-operative tracheostomy removed.

### VANCOUVER syndrome

Sensory fibers of the vagus nerve innervate the tracheobronchial tree and generate a “tickling” sensation (not pain) when activated. A vascular compression of this component of the vagus nerve can trigger coughing. This is similar to trigeminal neuralgia from compression of the sensory fibers within the trigeminal nerve except the sensation is a tickle not pain and the location is in the lungs not the face. The syndrome was named to reflect its etiology and as an homage to the location of its discovery. Vagus Associated Neurogenic Cough Occurring due to Unilateral Vascular Encroachment of its Root (VANCOUVER syndrome) can be diagnosed by a combination of history, physical examination and special tests (2).

#### History

Patients present with a medically refractory, chronic, dry cough. They describe an intermittent tickling sensation that triggers an irresistible cough. The sensation (and resultant cough) can be aggravated by prolonged or loud talking, harsh fumes or an upper respiratory infection and sometimes has a positional component (i.e., lying down on one side may worsen or ease the symptoms). The sensation and cough can occur while asleep and will awaken the patient. Patients should be seen by a laryngologist or respirologist to rule out postnasal drip, asthma, gastroesophageal reflux, infection, chronic obstructive lung disease, side effect from angiotensin-converting enzyme inhibitors, aspiration, bronchiectasis, bronchiolitis, cystic fibrosis, lung cancer, sarcoidosis, idiopathic pulmonary fibrosis or CANVAS. The symptoms will slowing worsen over the years but may have months of remission like trigeminal neuralgia. The medications used for trigeminal neuralgia can be effective for this vagal sensory pathology.

#### Physical

In between episodes, the patient’s examination is entirely normal. As with hemi-laryngopharyngeal spasm, the temporal juxtaposition of a patient with a severe, debilitating cough who, moments later, is entirely normal often leads to the misdiagnosis of a psychogenic disorder. Their cough is a normal brainstem response to an abnormal sensory stimulus from their vagus nerve. Their examination is only remarkable for the lack of any findings suggestive of another pathology.

#### Special tests

**Figure 4:**
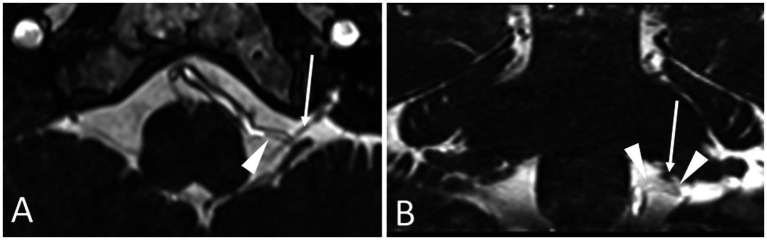
Axial **(A)** and coronal **(B)** MRI showing the PICA (arrowhead) encroaching the vagus nerve (arrow).

MRI must demonstrate a vascular compression of one of the vagus nerves. Imaging sequences are the same as for the other neurovascular compression syndromes (CISS or FIESTA). As with hemi-laryngopharyngeal spasm, care must be taken not to over diagnose the condition because asymptomatic patients can have a vessel on their vagus nerve up to 40% of the time (6). An MRI documented compression of the vagus nerve is therefore required but not sufficient for the diagnosis of VANCOUVER syndrome. If there is no compression, the diagnosis can be excluded. An example of an MRI demonstrating the posterior inferior cerebellar artery (PICA) encroaching the vagus nerve is shown in Figure 4.

We have been using inhaled lidocaine (5 cc of 2% in saline) as a screening test. As with trigeminal neuralgia, anesthetizing the affected area provides a temporary relief. The affected area for VANCOUVER syndrome is somewhere within the vagus-innervated tracheobronchial tree. Nebulized lidocaine is often used by laryngologists to provide a local anesthetic for minor operations in that region and more recently to treat chronic cough (7). If inhaled lidocaine stops the tickling sensation and cough, then we proceed to more definitive tests (see below). If it does not, the diagnosis is excluded.

The definitive diagnostic test for VANCOUVER syndrome is resolution of symptoms followingly a unilateral vagus nerve block. If a vagus nerve block ipsilateral to the vascular compression shown on MRI stops the cough and a vagus nerve block on the contralateral side does not, then the diagnosis of VANCOUVER syndrome is confirmed. We have been completing the contralateral (negative control) test to ensure this is not a generalized problem. If the patient’s cough stops when only when one side of their vagus nerve is temporarily blocked then the condition is clearly unilateral. The many conditions that can cause chronic cough (noted above) are unlikely to be resolved with a unilateral treatment. If that side is also the side with an obvious vascular compression of the vagus nerve at the brainstem, then it is likely the cause of their cough and warrants a discussion about the pros and cons of a surgical cure.

**Figure 5:**
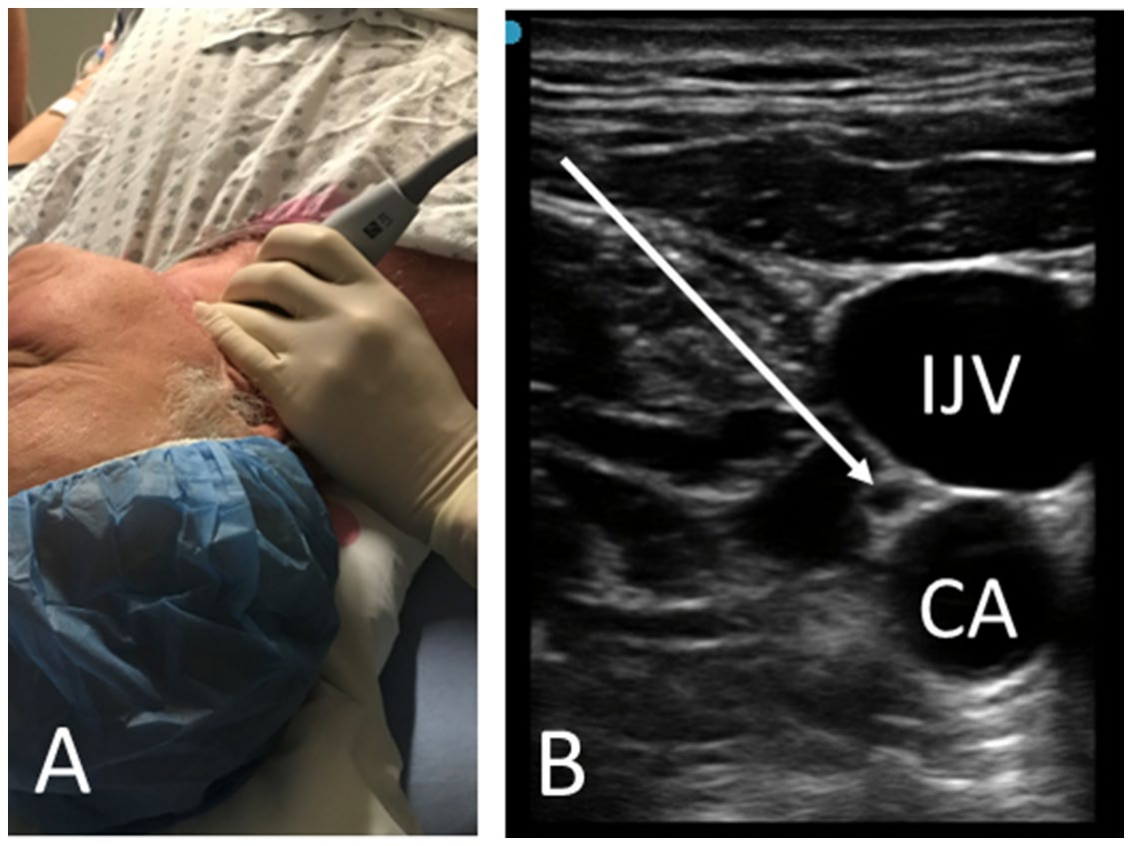
Ultrasound technique **(A)** and image **(B)** showing vagus nerve and direction of needle (arrow), carotid artery (CA) and internal jugular vein (IJV).

The vagus nerve block is performed by our anesthetist who has experience with nerve blocks. The vagus nerve can be seen in the neck with ultrasound guidance (Figure 5) and can be temporarily anesthetized with lidocaine (4 cc of 2%). The vagus nerve travels with the carotid artery (which pulsates) and internal jugular vein (which can be compressed) during ultrasound imaging. When the vagus nerve is anesthetized, there will be a unilateral vocal fold palsy causing a hoarse voice. During the time the voice is hoarse, the patient’s tickling sensation and cough should resolve. When the anesthetic wears off, the voice will return to normal and the cough should begin again. This would be a positive test. When the vagus nerve block is performed on the contralateral side, there should be a temporary hoarse voice but no affect on the cough. Any attempted vagus nerve block that does not cause a hoarse voice was unsuccessful (the vocal fold paralysis and resultant hoarse voice is the positive control for the successful vagus nerve block).

Although vagus nerve block has been described before, it is not a common procedure and should be approached with care (8, 9). Ultrasound guidance has improved the targeting of the nerve and our tests are performed in the operating room by an anesthesiologist with expertise in regional blocks. The much more familiar superior laryngeal block (10) does not sufficiently cover the tracheobronchial tree to be used for this test.

#### Diagnostic protocol

**Figure 6:**
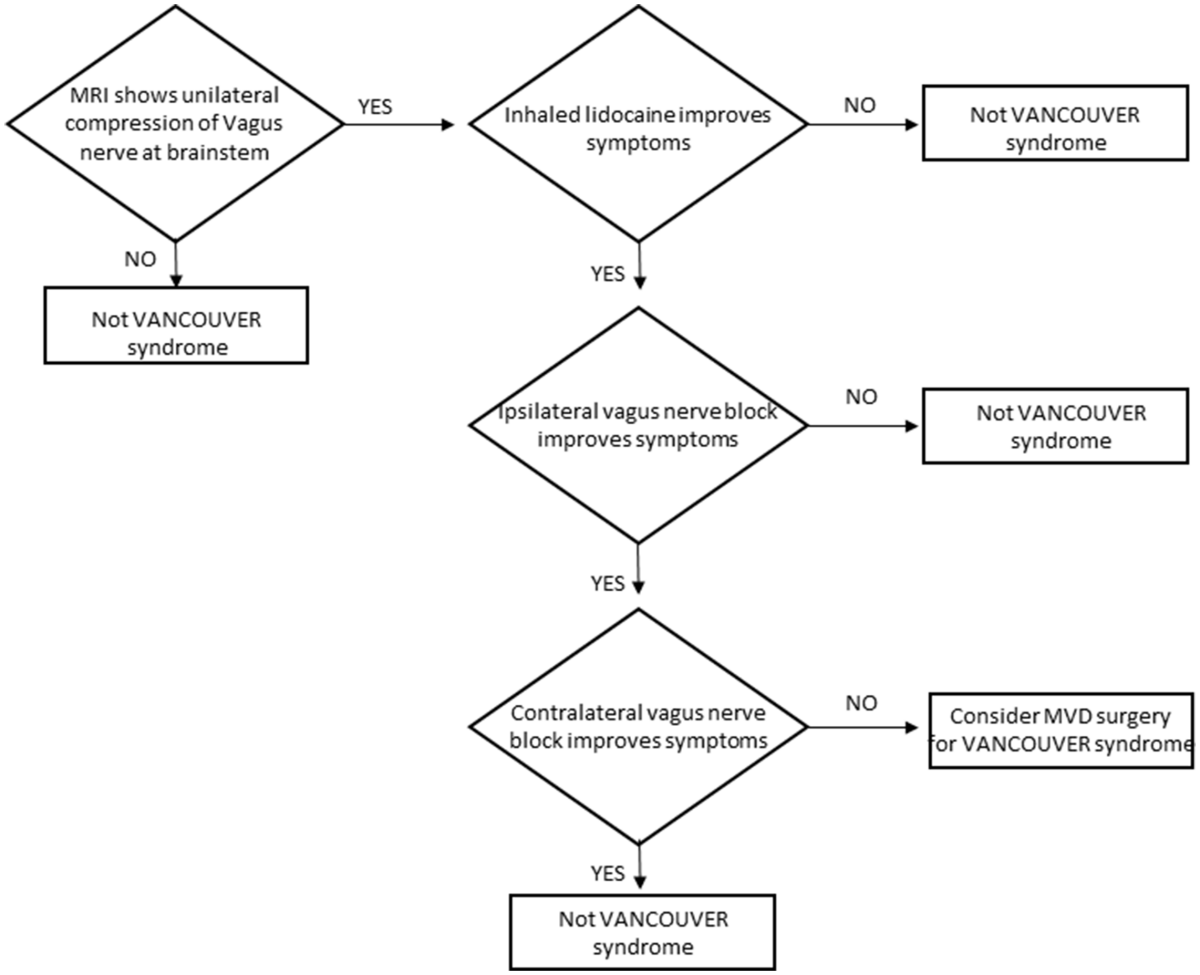
Diagnostic protocol for VANCOUVER syndrome.

Patients with neurogenic cough refractory to all medical investigations can be tested for a diagnosis of VANCOUVER syndrome. The diagnostic protocol for patients is shown in Figure 6. Patients may have a response to anti-neuralgia medications but all investigations and other therapies will have failed. The initial screening test is an MRI. If there is no vascular encroachment of the vagus nerve, then the diagnosis of VANCOUVER syndrome can be excluded. If there is a unilateral vascular encroachment of the nerve, then proceed to the secondary screening test – inhaled lidocaine. If anesthetizing the tracheobronchial tree temporarily stops the cough then proceed to the definitive test – vagus nerve block. If the inhaled lidocaine does not stop the cough, the diagnosis can be excluded.

The definitive test is invasive and requires experience in ultrasound-guided nerve block. If unilateral vagus nerve block ipsilateral to the MRI-demonstrated vascular encroachment of the vagus nerve temporarily stops the cough and contralateral vagus nerve block does not stop the cough, the diagnosis of VANCOUVER syndrome is confirmed and warrants discussion of the pros and cons of a potential surgical cure.

#### Medical therapy

The vagus-mediated tickling sensations can be diminished with anti-neuralgia medications. This is similar to trigeminal neuralgia. The side effects of these medications (e.g., Carbamazepine, Neurontin) are well known and can include rash, sedation and cognitive blunting. For patients who cannot stop their coughing with medications or cannot tolerate the side-effects of the medications required to stop this coughing, surgery may be an option.

#### Surgical therapy

**Figure 7:**
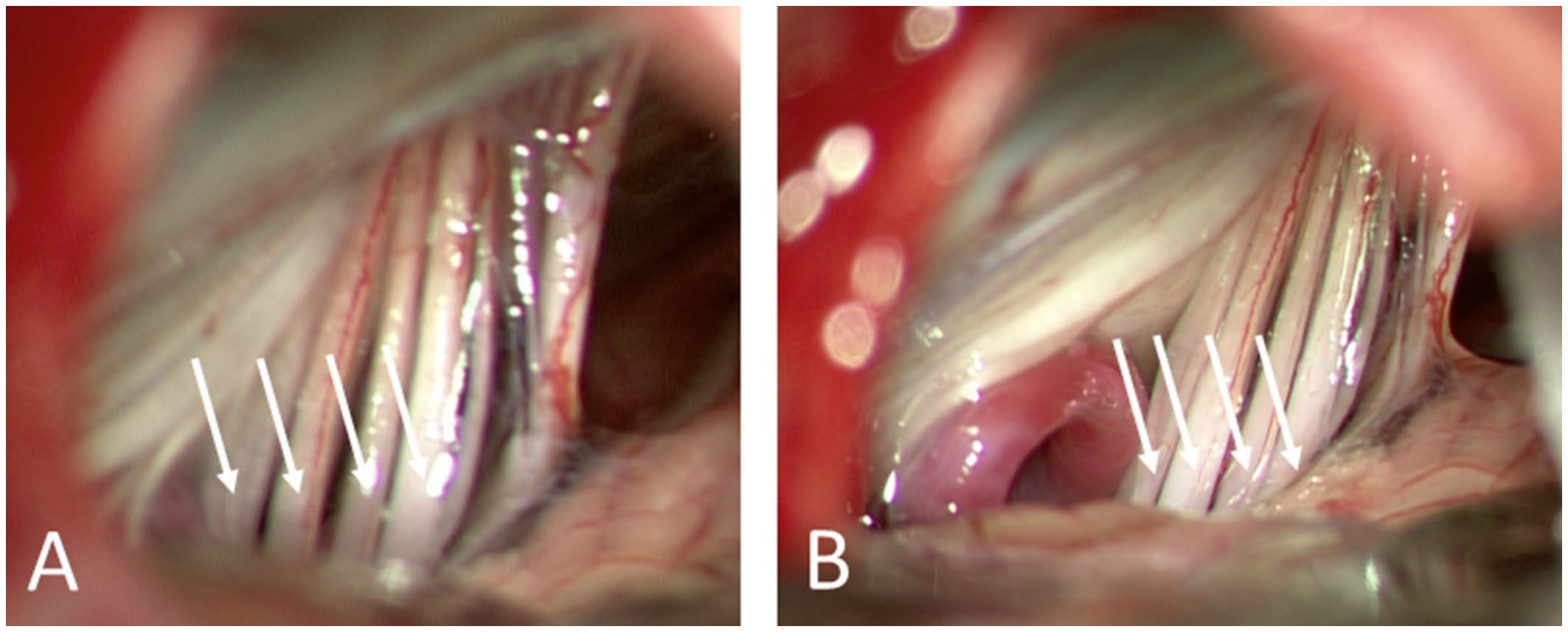
Microscopic view of four rootlets of the vagus nerve (arrows) before **(A)** and after **(B)** decompression from a loop of the PICA that was encroaching on the nerve from anterior and displacing them posterior.

The definitive therapy for VANCOUVER syndrome is microvascular decompression of the vagus nerve. The surgical approach is identical to that for hemi-laryngopharyngeal spasm describe above. An example of the intraoperative imaging seen before and after decompression of the vagus nerve is shown in Figure 7. The vagus nerve rootlets were displaced posterior toward the microscope by a loop of the PICA. When the loop was rotated 90 degrees, it passed between the vagus and accessory nerves.

The benefits are typically seen immediately. There is a relatively unique potential complication with any residual Teflon left against the lowest rootlet of the vagus nerve – delayed dysphagia. We recommend nothing be left pressing against the vagus nerve; not the vessel nor the Teflon padding.

## Concurrent glossopharyngeal neuralgia and HELPS or VANCOUVER syndrome

The anatomical juxaposition of cranial nerves IX and X makes the possibility of concurrent symptoms possible. We have published such a case (5). The presence of unilateral pain characteristic of glossopharyngeal neuralgia makes the diagnostic work-up easier – it is clear what side the pain is on and surgery can be considered if the symptoms are medically refractory. The Rushton et al. case series of patients with glossopharyngeal neuralgia (*n* = 217) reported that the majority of those patients had pain in the characteristic location and pattern without any additional features. Some of those patients, however, reported glosspharyngeal neuralgia with coughing (*n* = 18) and few with stridor or hoarseness (*n* = 3) (11). We postulate that those patients had glossopharyngeal neuralgia and VANCOUVER syndrome (pain with cough) or glossopharyngeal neuralgia with HELPS (pain with hemi-laryngopharyngeal contractions) before the authors knew the conditions existed. The Bohm and Strang series of 18 patients with glossopharyngeal neuralgia treated in Sweden included 2 with the additional symptoms of coughing (12). One of those patients (case 11 from 1947) described her symptoms as, “attacks of severe cramps in the throat, a feeling of suffocation and dyspnoea” in addition to her repeated 15 s bouts of burning pain in the tonsil radiating to her ear. We postulate that she was describing glossopharyngeal neuralgia and HELPS because glossopharyngeal neuralgia alone does not present with throat contractions. These historical reports of patients with additional symptoms (cough or throat contractions) in addition to their glossopharyngeal neuralgia have been found in at least 4 languages and multiple countries (5).

## Current issues

The final widespread acceptance of these conditions will require their recognition and treatment by multiple centers. At the present time, all the peer-reviewed publications on these two condition have come from only one group in Vancouver and a single publication from Japan (13). This lack of confirmation from well established neurosurgical centers of excellence around the world continues almost a decade after the initial description of HELPS in the Journal of Neurosurgery. We believe this is partial due to the initial publications being presented in the neurosurgical literature instead of the otolaryngoogy or respirology literature. Neurosurgeons will be needed to cure the condition but will never see a patient until they are referred to them. These patients typically present to our otolaryngology and respirology colleagues (or emergency physicians) and are often misdiagnosed with a psychogenic disorder and referred to psychiatry. Additional work is needed promoting the diagnosis of these conditions within the otolaryngology and respirology communities.

## Future issues

The next step may be the recognition of a third vagal rhizopathy. The vagus nerve carries motor, sensory and parasympathetic fibers (14, 15). The clinical ramifications of compression of the motor (hemi-laryngopharyngeal spasm) and sensory (VANCOUVER syndrome) components have been described above. What about the parasympathetic component?

At the present time, we do not have definitive proof of a parasympathetic vagus rhizopathy but have accumulated data that points to the condition. We postulate that compression of the vagus nerve parasympathetic fibers leads to a reduction of parasympathetic function. This would be analogous to compression on the 3rd cranial nerve’s parasympathetic fibers causing a pupillary dilation due to imbalance with sympathetic innervation. The vagus nerve innervates the lower esophageal sphincter and facilitates its opening during swallowing. The sphincter’s sympathetic innervation facilitates its closure to prevent acid reflux. We postulate that compression of the vagus nerve parasympathetic fibers would reduce the parasympathetic innervation of the lower esophageal sphincter and the resultant imbalance of sympathetic innervation would make it difficult to open this sphincter during a swallow. The clinical result would be dysphagia.

There is a recently described condition, esophagogastric junction outlet obstruction (EGJOO), that has many features in common with what would occur if the lower esophageal sphincter had reduced parasympathetic innervation. One of our patients developed a dyspahia four years post-operatively. Esphagogastric manometry was consistent with dysfunction of the lower eosphagogatric sphincter. Their symptoms resolved following a repeat MVD that decompressed the lowest rootlet of the vagus nerve from the Teflon mass. Future studies will determine if the cause of this currently idiopathic condition is the third vagal rhizopathy. Interestingly, we have not recognized any cardiac issues post-operatively (tachycardia).
